# Traditional Chinese and Indian medicine in the treatment of opioid-dependence: a review

**Published:** 2013

**Authors:** Fatemeh Doosti, Saeedeh Dashti, Seyed Meghdad Tabatabai, Hossein Hosseinzadeh

**Affiliations:** 1 *School of Pharmacy, Mashhad University of Medical Sciences, Mashhad, I. R. Iran*; 2 *Pharmaceutical Research Center, Department of Pharmacodynamics and Toxicology, School of Pharmacy, Mashhad University of Medical Sciences, Mashhad, I. R. Iran*

**Keywords:** Addiction, Opioid Tolerance, Opioid Withdrawal, Traditional Chinese Medicine, Traditional Indian Medicine

## Abstract

**Objective**: In this study, the current literatures on the use of herbs and herbal preparations of Traditional Chinese and Indian Medicine for the treatment of opioid addiction were reviewed**.**

**Matherials and Methods**: Search was done in databases such as Pub Med, Science Direct, Scopus, Springer Link, and Google Scholar.

**Results**: Among 18 retrieved studies, 3 studies were about asafetida extract, an approved preparation for ameliorating drug abstinence in China. Chinese preparations including Composite Dong Yuan Gao, Qingjunyin and TJ-97 (a water extract of dai-bofu-to) as well as Indian ones, Mentate and Shilajit, were reported to have positive effects against opioid withdrawal, dependence, and tolerance. Moreover, Levo-tetrahydropalmatine and L-Stepholidine, in addition to extracts of *Caulis Sinomenii *and *Sinomenium acutum *showed similar effects.

Banxia Houpu Decoction, Fu-Yuan pellet, Jinniu capsules, Qingjunyin, Tai-Kang-Ning capsule, and Xuan Xia Qudu Jiaonang (WeiniCom) from Chinese preparations, showed anti-addiction effects in randomized, double-blind and, in some studies, multicenter clinical trials.

**Conclusion** *:* Traditional herbal preparations of China and India have anti-addiction effects with less adverse effects than alpha2-adrenergic or opioid agonists.

## Introduction

Opioids are important drugs for relieving severe pain. Unfortunately, developing tolerance and dependence to them are problems associated with their use. Tolerance causes a need to increase the dosage and dependence induces withdrawal syndrome if the drug is discontinued (Christie 2008 ▶). Furthermore, there is no approved treatment for opiate tolerance and dependence. To find a proper treatment, herbal therapy, complementary, and traditional medicine are extremely appreciated. Herbal preparations of Chinese and Indian traditional medicines and some plants belonging to these medicine systems with effect on opioid tolerance and dependence are reviewed in this study.

Traditional Chinese Medicine (TCM) has been applied in China for over two thousand years and has been used for treatment of drug addiction for 200 years. At least 10 medicines of TCM have been approved for the treatment of opiate addiction by Chinese State Food and Drug Administration (SFDA) (Shi et al., 2006 ▶). 

## Method

We searched articles about the remedies in traditional Chinese and Indian medicines for treatment of opioid addiction. The search was done in databases such as PubMed, ScienceDirect, Scopus, Springer Link and Google Scholar. The articles were studied and their main points were extracted and mentioned.

## Results

Several preparations, plants, and active ingredients of plants were found in this search with certain effects on opioid addiction proven in animal or clinical studies. These include:

**Asafetida extract**

Asafetida extract is derived from factice resin of *Ferula sinkiangensis K. M. Shen* or *Ferula fukanensis K. M. Shen*. Some of its ingredients are a-pinene, 2-borneol, terpin-4-ol, D-fenchyl alcohol, pinocarveol, disulfide compounds, 2, 2-dimethylthiopropane, 1, 2-diethylthiopropane and N, N-dimethylthioformamide. It is effective in destroying intestinal worms, treatment of parasite-induced malnutrition, swelling pain in stomach and abdomen, abdominal mass, cold, malaria, diarrhea, and prevention of measles, but its strong odor has limited its usage (Wang, 2007 ▶)

It has also been used in a pharmaceutical preparation which is useful in ameliorating drug abstinence and treatment of addicted subjects to opioids, marijuana, diamorphine, and the like. Its bitter taste and strong odor was weakened or covered up by new techniques (Wang, 2007 ▶).In the experimental stage, 50 rats were made dependent to morphine in 14 days. Ten other rats received saline at dose of 0.2 ml/100 g according to the times of morphine injections, as negative control group. On the 13^th^ day, the day before the last morphine injection, the 50 rats were divided into 5 groups including morphine-dependent group, positive control group which received clonidine perfusion and 3 groups receiving 4, 2, and 1 g/kg of asafetida injection (i.p.) on days 13 and 14. Negative control group and morphine-dependent group received only saline in these 2 days. 

Animals were treated with a last dose of morphine on day 14, one h after receiving clonidine or the extract, animals were treated with morphine. Forty minutes later, they were weighed and injected by naloxane (4 mg/kg). Abstinence symptoms such as abnormal posture, irritation, teeth chattering, and autonomic nervous system symptoms such as lacrimation, diarrhea, and salivation of rats were observed and recorded for 2 h. Body weights of rats were measured at 30 and 60 min after naloxane injection.Results showed that asafetida injection could significantly decrease scores of abstinence reactions and inhibited the weight loss compared with morphine-dependent rats (Wang, 2007 ▶).

In the clinical stage, drug-addicted people were randomly divided into asafetida-injected group (thrice per day, 2 ml of the extract, equivalent to 1 g of original crude material per time, intramuscularly, for 3 days), Phenoxyimidazoline hydrochloride group (0.1 mg/tablet, thrice per day, a tablet per time, for 3 days) as positive control group and negative control group (thrice per day, 2 ml saline per time, intramuscularly, for 3 days). Abstinence symptoms were evaluated. 

As a typical case, subjects addicted to heroin for over 10 years were chosen and the same clinical trial was done for them. The only difference was that asafetida-injected group received 5 ml of the extract (equivalent to 2.5 g of original crude material) per time. Moreover, gooseflesh, chills, flaccidity, general aching, and muscular twitching were observed in addition to the mentioned symptoms. These two clinical trials showed that this extract had ameliorating effects on both "moderate" and "serious, deeply, over a long time addiction to drugs". This medicine is available in various dosage forms including injection, capsules, drop pill, tablet, granule, powder, oral liquid. These are explained in detail in the patent with exact pharmaceutical methods to prepare them (Wang, 2007 ▶).

**Xuan Xia Qudu Jiaonang (WeiniCom)**

Xuan Xia Qudu Jiaonang (WeiniCom) is a herbal compound in Chinese medicine. It is composed of extracts of *Coptis chinensis* rhizome*, Schisandra chinensis, Angelica sinensis, Astragalus membranaceus, Glycyrrhiza glabra, Terminalia chebula, Zingiber officinalis, Ziziphus jujube, Panax quinquefolius, Ganoderma Lucidum, Nauclea* Spp.*, Cordyceps sinensis, Magnolia officinalis, Epimedium grandifora, Corydalis yanhusuo, Corydalis decumbentis, Eleutherococcus senticosus, Sida Cordifolia,* and* Rhodiola crenulata *(Hao and Zhao, 2000 ▶). 

 In a double-blind clinical trial, the effect of this compound on withdrawal signs and craving was investigated. Forty-two heroin addicts were randomly divided into two groups, 21 cases in WeiniCom group and 21 cases in buprenorphine group. There was no negative control in this study. During 14 days of treatment period, withdrawal signs and craving and also the adverse effects were evaluated in these two groups. Treatment in both groups was safe and well-tolerated but the reduction in withdrawal symptoms and craving was better and faster in WeiniCom group. WeiniCom needed a shorter period of time to make an effect (Hao and Zhao, 2000 ▶). 

**Qingjunyin** **(QJY)**

Qingjunyin (QJY) is a Chinese herbal preparation and its effect on withdrawal syndrome was evaluated both experimentally and clinically. In the experimental study, white rats and mice became dependent on morphine by increasing doses of morphine. Afterward, they were divided into 4 groups, large dosage QJY group, small dosage QJY group, sustained morphine group, and control group. After naloxane injection to the animals, number of jumping reflexes was reduced and animal weights were enhanced significantly and dose-dependently in QJY groups (Lu et al., 1998 ▶).

In a clinical study, 100 heroin addicts received 180 ml QJY (p.o.) once a day for 10 days as QJY group. Two groups, including 50 cases in each group, were treated with clonidine or methadone for 10 days as positive control groups. There was no placebo group in this study. The effect of QJY on treatment of addiction was observed according to detoxification standards. The abstinence symptoms in QJY detoxification group were lower than clonidine group in the first 3 days of the trial, but later, this difference was not seen and it was as effective as clonidine. The scores of abstinence syndrome were equal in QJY and methadone groups in the first 5 days of the treatment but in the late 5 days of the treatment, QJY was less effective than methadone. (Lu et al., 1997 ▶).

**Jinniu capsules**

Jinniu capsules contain herbs and marine products extracts used in traditional Chinese medicine. In a randomized, double-blind and multicenter clinical trial, the efficacy and safety of Jinniu capsules in the treatment of heroin withdrawal symptoms were investigated in comparison with lofexidine. Two hundred and twelve heroin addicts were divided into 2 groups and were treated with Jinniu capsules or lofexidine for 10 days. There was no negative control in this study. The withdrawal symptoms were assessed daily and anxiety scores were evaluated on days 0, 5, and 10. Vital signs and side effects were also checked to assess the safety of Jinniu capsules. Withdrawal signs and anxiety scores decreased over the time of the trial in both groups and there was no significant difference between them. Moreover, no serious adverse effects were observed in the treatment period (Shi et al., 2008 ▶).

**Levo-tetrahydropalmatine (l-THP)**

Levo-tetrahydropalmatine (l-THP) is an alkaloid present in plenty of Chinese herbal preparations. It is obtained from Chinese herbs of Corydalis and Stephania genera. In a study, the inhibitory effect of l-THP on psychological dependence and locomotor stimulation induced by oxycodone was shown by conditioned place preference (CPP) method in rats and mice. In this study, oxycodone treatment (0.32-5.0 mg/kg) resulted in the development of CPP and oxycodone (2.5 mg/kg) enhanced cAMP response element-binding (CREB) and extracellular signal-regulated kinases (ERK) phosphorylation in nucleus accumbens and hippocampus. l-THP at doses of 6.25-18.50 mg/kg did not show any CPP or aversion solely but co-treatment with l-THP and oxycodone in conditioning phase reduced the development of CPP induced by oxycodone in rats. It also inhibited the increase of CREB and ERK phosphorylation in nucleus accumbens and hippocampus which may be the underlying mechanism in inhibition of oxycodone- induced CPP (Liu et al., 2009b ▶).

**L-Stepholidine**

L-Stepholidine, an alkaloid found in Chinese herbs of *Stephania *genus. L-Stepholidine (SPD), is a D1 receptor partial agonist and D2 receptor antagonist. It has been proven to have antipsychotic effects in animal models. In a study from 2007, the effect of SPD on CPP induced by morphine was investigated in rats. CPP was induced in rats by daily injection of morphine (10 mg/kg, i.p.) for 6 days. Daily administration of SPD before morphine injection within these 6 days decreased morphine induced CPP, in a dose-dependent manner (acquisition test) but a single treatment with morphine (10 or 20 mg/kg, i.p.) on the day following acquisition of morphine did not have any inhibitory effect on CPP induction at any of the doses (expression test). CPP disappeared after 21 days of saline-treatment. After that, a single morphine injection (3 mg/kg, i.p.) resulted in re-acquisition of morphine induced CPP. However, administration of SPD (10 or 20 mg/kg), 30 min before this 3 mg/kg dose of morphine, inhibited morphine CPP re-acquisition. It was also shown that SPD did not show any effect on food-induced CPP so it does not act through the mechanisms related to learning ability (Wang et al., 2007 ▶). 

**Banxia Houpu Decoction**

Banxia Houpu Decoction contains *Pinellia ternata*, *Poria cocos*, *Magnolia officinalis*, *Perilla frutescens,* and *Zingiber officinale*. It has been used for treatment of diseases related to depression for hundreds of years in China (Rauf et al., 2012 ▶). 

The efficacy of Modified Banxia Houpu Decoction (MBHD) in treatment of protracted heroin withdrawal symptoms was studied in a randomized clinical trial. In this study, 187 heroin addicts were divided into 3 groups, 58 addicts in control group, 62 in treatment group A and 67 in treatment group B. Detoxification was done by lofexidine hydrochloride tablet in all the addicts during 12 days. Treatment group A received MBHD since the end of the detoxification period but group B received it from the beginning of the detoxification for 60 days. Ten days after MBHD treatment, protracted withdrawal symptoms were observed and re-abusing rate in patients after 1 year was measured by urine test. The score of protracted symptoms of withdrawal syndrome was decreased in treatment groups compared with the control group. Moreover, Group B showed a significantly better response in comparison with group A. Re-abusing rate was also significantly less than control group and group A. MBHD was then concluded to be useful to ameliorate protracted withdrawal symptoms in heroin addicts after detoxification (Huang et al., 2004 ▶).

**Fu-Yuan pellet (FYP)**

Fu-Yuan pellet (FYP) is a formula in TCM for detoxification of opiates. It consists of 10 herbs, including Divaricate Saposhnikovia Root, Glabrous Greenbrier Rhizome, Prepared Dried Ginger, Dried Tangerine peel, Excrementum Pteropi, Hawthorn Fruit, Eucommia Bark, Desertliving Cistanche, Tangshen, and Largehead Atractylodes Rhizome (Mesh). In a randomized and double-blind clinical study, the efficacy and safety of FYP for treatment of heroin addiction was investigated. In this study, 225 heroin addicts, aged 18-55, were chosen according to Diagnostic and Statistical Manual of Mental Disorders, Fourth Edition (DSM- IV) classification criteria such as positive response in urinary heroin test 8-36 hours after the last time of heroin usage and indicating withdrawal syndrome scores above 50. This multicenter study was done in 3 drug-addiction treatment centers in China. Lofexidine was used as a positive control. Subjects were treated with FYP or lofexidine in a similar schedule for 10 days and total withdrawal scores and daily rate of reduction in withdrawal syndrome signs were measured daily. Both groups showed a significant reduction in withdrawal signs after 3 days and also FYP had fewer side effects than lofexidine (Wang et al., 2009 ▶).

**TJ-97**

TJ-97 is a water extract prepared from dai-bofu-to which is a Kampo medicine (Chinese traditional prescriptions composed of herbal drugs).

The effect of TJ-97 on withdrawal syndrome was assessed on isolated ileum segments of guinea pigs which were dependent on morphine. After naloxone administration, TJ-97 reduced contractions in the ileum segments in the presence or absence of atropine. It also decreased contractions induced by low-frequency electrical stimulation or nicotine in the segments but it did not inhibit the contractile responses resulted from exogenous Acetylcholine or substance P. Morphine-dependent rats were also treated with TJ-97(i.p.) and after 30 min received naloxone. These rats had less number of excretions and less amount of feces or diarrhea. Therefore, it was claimed that TJ-97 acts through inhibition of the release of Ach and substance P from nervous structures in ileum wall (Takamura et al., 1995 ▶).

**Tai-Kang-Ning (TKN) capsule**

Tai-Kang-Ning (TKN) capsule is an herbal formula in TCM. In a randomized and double-blind clinical study, the efficacy and safety of TKN for treatment of acute heroin abstinence symptoms were evaluated. Sixty-four heroin addicts with acute withdrawal symptoms were chosen and were treated with lofexidine, as a positive control, or TKN in a similar schedule for 10 days. Both groups showed reduction in acute withdrawal symptoms after 3 days and there was no significant difference in the efficacy and safety between 2 groups (Kang et al., 2008 ▶).

**Composite Dong Yuan Gao (CDYG)**

Composite Dong Yuan Gao (CDYG) is a Chinese herbal preparation. Its effect on morphine withdrawal symptoms was studied in white rats and mice. Animals became dependent on morphine by increasing doses of morphine and then were divided into 4 groups, small dose CDYG group, large dose CDYG group, sustained morphine group, and control group. After naloxan injection (i.p.), number of jumping responses was reduced and animal weights were enhanced significantly and dose-dependently in CDYG groups than control group (Wu et al., 1995 ▶).

***Caulis Sinomenii***

Caulis Sinomenii is a Chinese plant and is known for its anti-inflammatory effects. The effect of this plant (10 g/kg) and sinomenine (60 mg/kg) on morphine CPP was assessed in mice. Histamine levels were also measured in the mice brains. Treatment with the extract as well as sinomenine reduced morphine place preference and lowered histamine levels in the brain (Mo et al., 2006 ▶).

***Camellia sinensis***

Green tea or *Camellia sinensis* (Theaceae) is a very popular herb used all over the world. The effects of (-)-epigallocatechin gallate, one of the main tannins was investigated in morphine-dependent rats. This compound attenuated withdrawal signs dose-dependently. It also showed inhibitory effects on morphine-induced increased cAMP concentrations in the locus coeruleus at 100 mg/kg. Moreover, it inhibited D-2 dopamine receptor signaling (Oh et al., 2007 ▶).

**Mentate**

Mentate is a proprietary psychotropic preparation made of eight Indian herbal drugs common in ayurvedic medicine: Brahmi (*Hydrocotyle asiatica*), Shatavari (*Asparagus racemosus*), Buchh (*Acorus calamus*), Ashwagandha (*Withania somnifera*), Giloi (*Tinospora cordifolia*), Amla (*Emblica officinalis*), Shankhpushpi (*Evolvulus alsinoides*), Kuth (*Saussurea lappa*), and Triphala. In a study, the effects of chronic administration of this preparation on morphine tolerance and dependence were investigated in Balb-C mice. The mice were pretreated with saline or Mentate at various doses (20-500mg/kg s.c.), and after 30 minutes, morphine (10 mg/kg) or saline was injected (twice a day). The analgesic responses were tested on days 1, 3, 9, and 10 by the tail flick test. Moreover, naloxone-precipitated jumping response was assessed. The results showed a significant inhibition of tolerance to the analgesic effects, with tail flick latency response reaching the cut-off point (10 seconds) with high doses of Mentate on days 9 and 10. 

The drug had no intrinsic analgesic property since the tail flick latency score of Mentate-treated groups were not significantly higher than saline-treated groups. Moreover, it was shown that the presence of the drug is not necessary when the test is being carried out, possibly ruling out any potentiating interaction of the drug with morphine. Furthermore, Mentate was shown to significantly and dose-dependently inhibit jumping responses after naloxone challenge. Still, it could not completely vanish these signs (Kulkarni and Verma, 1992 ▶).

**Shilajit**

Shilajit is a brownish exudate that emerges from decomposition of plant and animal material by the pressure of layers of rock. This ayurvedic drug has long been used as an adaptogen and rejuvenator in ancient Indian medicine. Many medical applications have been proposed for Shilajit and its active constituents (namely humic acid and fulvic acid) but there have not been plenty of convincing data to prove these properties (Emami et al., 2012 ▶; Innocenti et al., 2007 ▶). Nonetheless, in one study in 2001, processed Shilajit was shown to inhibit tolerance to analgesic effects of morphine. Swiss mice were injected with morphine (10 mg/kg, i.p., twice a day) for 10 days. The control group received vehicle on the same schedule. Two groups (n=8) were administered 0.1 and 1 mg/kg of processed Shilajit, from day 6 through day 10. On day 11, all groups received 10 mg/kg i.p. of morphine and the analgesic response was measured by the hot plate test. The mice were tested every 30 minutes up to 240 minutes. The drug, with both doses, reduced significantly the tolerance to analgesic effects of morphine. Shilajit by itself, had no analgesic effects at the administered doses, nor did it potentiate the analgesic effects of morphine, as morphine-naïve mice treated with processed shilajit showed no significant difference compared with the control group. The authors attributed this effect to the immunomodulatory properties of Shilajit, but no test was done to prove that (Tiwari et al., 2001 ▶).

***Benincasa hispida Cogn***

*Benincasa hispida Cogn* ***.*** (Cucurbitaceae) fruits are popular vegetable in tropical regions such as India. The fruit is mentioned in the Ayurveda to be beneficial in nervous disorders. In animal models, its analgesic effect has been demonstrated. It contains triterpenes, sterols, and glycosides. 

Grover et al. (2000) ▶ evaluated the fresh fruit pulp juice of this plant for its preventive and suppressive potential against morphine withdrawal syndrome in morphine-dependent Swiss mice. Mice were grouped into saline, untreated dependent and treated (1 ml/mouse, p.o., along with morphine) dependent. The mice were observed for their jumping reflexes 16 h after the last dose of morphine. The untreated dependent group was then divided into two subgroups, one received 1 ml of the juice p.o. 17 and 27 h after the last morphine dose and the other received no treatment. The subgroups were then assessed for their jumping (for 2 min) and defecation reflexes (for 24 h), 29 h after the last dose of morphine. In the first part, the mice treated with *B. hispida juice *did not have jumping episodes compared with untreated dependent mice. In the second part of the study, the subgroup which received the juice showed no jumping compared with the other subgroup and markedly lower number of stools. It was concluded that the juice of *B. hispida* could potentially prevent morphine withdrawal problems or suppress these complications after their onset. No positive control was used in this experiment and the method of induction of dependence was not clear. Different doses and different routes of administration could also have been examined (Grover et al., 2000 ▶).

**Table 1:** A brief review on herbal preparations of Traditional Chinese Medicine for the treatment of opioid addiction.

**Preparation or compound**	**Method**	**Tested** **Doses**	**Possible Mechanisms**	**Authors and Year**
**Asafetida extract**	Experimental study: Observing withdrawal signs	Experimental study: 4,2, and 1 g/kg of asafetida injection (i.p.)		Wang (2007)
**Asafetida extract**	Clinical study: Elevation symptoms and mood in addictions	Clinical study: thrice per day, 2 ml of the extract (equivalent to 1 g of original crude material per time)		Wang (2007)
**Asafetida extract**	Same clinical study as mentioned above on addicted subjects to heroin for over 10 years	Five ml of the extract (equivalent to 2.5 g of original crude material) per time		Wang (2007)
**Banxia Houpu Decoction**	A randomized clinical trial on 187 heroin addicts with protracted withdrawal symptoms			Huang (2004)
**Composite Dong Yuan Gao (CDYG)**	Recording withdrawal jumpings and body weights of rats and mice			Wu (1995)
**Fu-Yuan pellet (FYP)**	A randomized and double-blind clinical study on 225 heroin addicts			Wang (2009)
**Jinniu capsules**	A randomized, double-blind and multicenter clinical trial			Shi (2008)
**Levo-tetrahydropalmatine (l-THP)**	CPP method	6.25-18.50 mg/kg	Inhibiting the increase of CREB and ERK phosphorylation in nucleus accumbens and hippocampus	Liu (2009)
**L-Stepholidine (SPD)**	Elevation the effect of SPD on CPP induced by morphine in rats	10 or 20 mg/kg	SPD is a D1 receptor partial agonist and D2 receptor antagonist	Wang (2007)
**Qingjunyin** **(QJY)**	Experimental study: Recording withdrawal jumpings and body weights of mice			Lu (1998)
**Qingjunyin** **(QJY)**	Clinical study on100 heroin addicts	180 ml QJY (p.o.) once a day		Lu (1997)
**Tai-Kang-Ning (TKN) capsule**	A randomized and double-blind clinical study on 64 heroin addicts with acute heroin abstinence symptoms			Kang (2008)
**TJ-97**	On isolated ileum segments of guinea pigs		Through inhibition of the release of Ach and substance P from nervous structures in ileum wall	Takamura )1995)
**Xuan Xia Qudu Jiaonang (Weinicom)**	A double-blind clinical trial on 42 heroin addicts			Hao (2000)

**Table 2:** A brief review on herbal preparations of Traditional Indian Medicine for the treatment of opioid addiction.

**Plant or preparation**	**Methods**	**Tested Doses **	**Possible Mechanisms**	**Active Ingredients in the Plant**	**Authors (year)**
**Mentate **	Tail-flick test, Naloxone-precipitated jumping	20-500 mg/kg(s.c.)	No intrinsic analgesia	-	Kulkarni (1992)
**Shilajit (an aurvedic exudates)**	Hot plate test	-	No intrinsic analgesia, Immunomodulatory effects.	Humic acid, Fulvic acid	Tiwari (2001)

## Discussion

Traditional medicine systems of different regions such as China and India may be a reasonable option for the treatment of opioid dependence and withdrawal. Useful and effective preparations from these medical systems were mixed compositions from several plants*. Zingiber officinale, Magnolia officinalis, Coptis chinensis, Astragalus membranaceus, Glycyrrhiza glabra, Ziziphus jujube *as well as* Ferula, Pinellia, Corydalis,* and *Stephania* genera were some of the main components of these preparations. Many plant extracts have been investigated for their inhibitory effects on opioid dependence (Kulisic et al., 2004 ▶; Hosseinzadeh and Jahanian, 2010 ▶; Hosseinzadeh et al., 2007 ▶; Lopes-Lutz et al., 2008 ▶; Hosseinzadeh et al.,2008 ▶). These studies may provide mechanistic basis for compound herbal preparations. On the other hand, traditional compounds may have the advantage of potentiated effects and multiple modes of action.

Clinical studies on this subject show promise. A meta-analysis done in 2009, which included 21 studies with 2949 cases in total, compared traditional Chinese herbal medicine with alpha2-adrenergic or opioid agonists for heroin detoxification. Chinese herbal medicines were better in ameliorating withdrawal symptoms score than alpha2-adrenergic agonists. However, Chinese herbal medicines were as effective as opioid agonists, though with a slower onset of action. Chinese herbal medicines had better efficacy than alpha2-adrenergic agonists in decreasing anxiety at the late stage of the treatment period but there was no significant difference in decreasing anxiety between Chinese herbal medicines and opioid agonists. Adverse effects such as fatigue or dizziness observed in traditional Chinese herbal medicines were significantly less than alpha2-adrenergic agonists. However, there are not enough data to compare the adverse effects between traditional Chinese herbal medicines with opioid agonists (Liu et al., 2009a ▶).

In some studies, mechanisms of the action of these preparations were investigated. These mechanisms were more various than chemical drugs which are mainly alpha2-adrenergic or opioid partial agonists. Some of the mechanisms include inhibition of the increase of CREB and ERK phosphorylation in nucleus accumbens and hippocampus, D1 receptor partial agonism and D2 receptor antagonism, inhibition of the release of Acetylcholine and substance P from nervous structures, and Immunomodulatory effects. These mechanisms may contribute to the fact that adverse effects such as dizziness and fatigue associated with these preparations tend to be milder than alpha2-adrenergics. Potential of dependencies was also less than opioid partial agonists.

In this review, we showed that Chinese plants, active ingredients of these plants, and Chinese and Indian preparations are as effective as chemical drugs for treatment of opioid addiction while having milder adverse effects. Therefore, identifying these native plants, extracting their components, and producing herbal preparations similar to these drugs by mixing these native plants can be helpful to find new ways for treatment of opioid addiction in the future.

## Conflict of interest

There is not any conflict of interest in this study.
